# Fabrication of Micro-Optics Elements with Arbitrary Surface Profiles Based on One-Step Maskless Grayscale Lithography

**DOI:** 10.3390/mi8100314

**Published:** 2017-10-23

**Authors:** Qinyuan Deng, Yong Yang, Hongtao Gao, Yi Zhou, Yu He, Song Hu

**Affiliations:** 1 State Key Laboratory of Optical Technologies for Microfabrication, Institute of Optics and Electronics, Chinese Academy of Sciences, Chengdu 610209, China; dqy_storm@163.com (Q.D.); gaohongtao@ioe.ac.cn (H.G.); alanzhouyi@163.com (Y.Z.); heyu@ioe.ac.cn (Y.H.); husong@ioe.ac.cn (S.H.); 2 University of Chinese Academy of Sciences, Beijing 100049, China

**Keywords:** maskless lithography, micro-optics elements, arbitrary surface, exposure dose, nonlinear effect

## Abstract

A maskless lithography method to realize the rapid and cost-effective fabrication of micro-optics elements with arbitrary surface profiles is reported. A digital micro-mirror device (DMD) is applied to flexibly modulate that the exposure dose according to the surface profile of the structure to be fabricated. Due to the fact that not only the relationship between the grayscale levels of the DMD and the exposure dose on the surface of the photoresist, but also the dependence of the exposure depth on the exposure dose, deviate from a linear relationship arising from the DMD and photoresist, respectively, and cannot be systemically eliminated, complicated fabrication art and large fabrication error will results. A method of compensating the two nonlinear effects is proposed that can be used to accurately design the digital grayscale mask and ensure a precise control of the surface profile of the structure to be fabricated. To testify to the reliability of this approach, several typical array elements with a spherical surface, aspherical surface, and conic surface have been fabricated and tested. The root-mean-square (RMS) between the test and design value of the surface height is about 0.1 μm. The proposed method of compensating the nonlinear effect in maskless lithography can be directly used to control the grayscale levels of the DMD for fabricating the structure with an arbitrary surface profile.

## 1. Introduction

During past decades, much research effort has been devoted to micro-optical elements with arbitrary surface profiles, which can usually achieve extraordinary properties far more than macro components, and has important applications in optical communication, sensors, special illumination, and other fields [1,2,3,4,5]. However, the limited fabrication methods for such micro-optical elements have restricted its development. Electron-beam lithography [6,7] and focused-ion beam [8] can realize a high-resolution fabrication of the structure with complicated surface profiles in principle, but require a long-term and expensive device. The direct laser writing technique [9,10,11] is a promising and economic method for the fabrication of microstructures, but the scanning mode will limit the improvement of work efficiency. The thermal reflow method [12,13,14] cooperated with conventional binary lithography is usually applied for micro lens array’s generation efficiently, but this method is difficult to control the surface profile precisely. Grayscale lithography [15,16,17] using a gray-tone mask is an effective method to obtain various exposure dose distributions on the photoresist by modulating the intensity of ultraviolet (UV) light. However, the physical gray-tone mask is usually fabricated by a direct writing lithography method and each grayscale mask can only be applied for a fixed structure. This is inconvenient for the flexible research of various structures and may lead to unnecessary cost.

Recently, maskless photolithography has been proposed for microstructure fabrication [18,19,20,21,22]. A digital micro-mirror device (DMD) has been adopted to replace the traditional physical mask. A DMD is composed of an array of micro-mirrors, each of which can be independently controlled by a computer, so a digital image acting as mask can be dynamically displayed in real-time. The great advantages of maskless photolithography are that no expensive physical mask is necessary. The digital image can be a flexible design according to the profile of the structure to be fabricated by computer. Due to its capability of low cost and high flexible, maskless lithography has received significant attention in the microfabrication field.

To date, the main approaches of fabrication art for achieving micro-optic elements based on the maskless photolithography are as follows: The first is to transfer the CAD data of the surface function of the structure to be fabricated into a serial slice along the direction of high, with each slice being a binary image. These binary images are generated by the DMD under computer control in real-time, and then are delivered by the imaging system to the surface of the photoresist where a superimposed exposure dose proportional to the profile function is obtained. Using this approach, Totsu et al. have fabricated the positive photoresist patterns of spherical and aspherical micro lens arrays with the diameter of each lens being 100 μm [23]. Zhong et al. also adopted the technique for the fabrication of continuous relief micro-optic elements [24]. Although these works can achieve the micro-optic elements at low cost with time savings, a common point of these methods is the tedious preprocessing of slicing for each design and multiple exposures are needed.

In this paper, we present a new fabrication art approach on the basis of maskless photolithography. By generating a grayscale map under an appropriate exposure time, a one-step exposure control for arbitrary surface profiles can be achieved, so both the slicing process and multiple exposures are avoided. The grayscale level is generated by the multiple reflection technique which adopts the means of pulse width modulation of the DMD. Although similar grayscale lithography based on DMD has been adopted by Wang et al. for the fabrication of diffractive optics [25,26], they do not describe the detailed experiment processing for the nonlinear effect existing in the fabrication procedure. In the digital grayscale lithography, a serious problem caused by DMD is that the exposure dose will not linearly change with respect to grayscale levels under a constant exposure time. Accurate general theoretical formulae for describing such nonlinear relationships are lacking. Additionally, the relationship between the exposure depth and exposure dose is also nonlinear due to the property of the photoresist. To compensate the two nonlinear effects and then to generate the appropriate grayscale map, we adopt the approach of calibrating the relationship between the exposure depth and grayscale levels under an appropriate exposure time that requires no specific knowledge about the nonlinear effects of the DMD and photoresist. During this procedure, a reasonable grayscale level range which maintains a smooth-slow increment and stable intensity distribution needs to be considered to ensure a well-controlled surface profile and suitable surface roughness. After generating the grayscale mask on the basis of the adjustment curve, a one-step exposure fabrication art has been built to obtain desired exposure depth. This method can ensure a precise control of the surface profile of the micro-optics element to be fabricated, and the process of slicing and multiple exposures are not needed.

To verify the reliability of this method, several typical array elements with a spherical surface, aspheric surface, and conic surface have been fabricated and tested, whose diameter is 200 μm and height is 6 μm. The photoresist molds were reversely replicated in polydimethysiloxane (PDMS) elastomer, which was widely used in micro-optics elements because of its excellent optical properties.

## 2. Experiment Setup and Methods

### 2.1. Maskless Photolithography System

**Figure 1:**
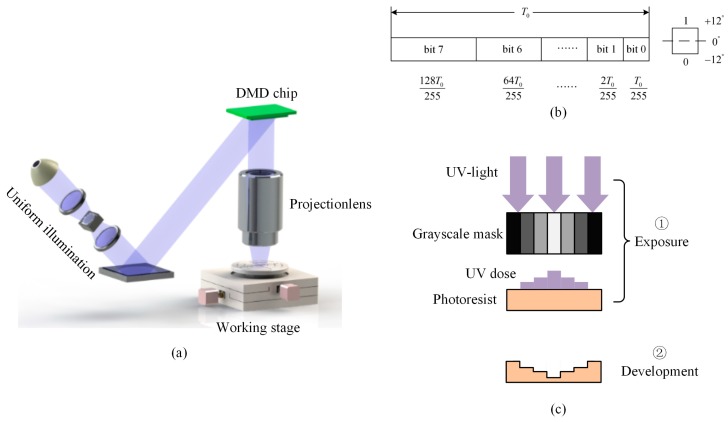
(**a**) Schematic view of the digital micro-mirror device (DMD)-based maskless photolithography system; (**b**) pulse width modulation based on 8-bit planes; and (**c**) the fabrication procedure based on grayscale mask exposure.

Figure 1a shows the maskless photolithography experimental setup in our laboratory, which consists of a uniform illumination system at 365 nm wavelength, a high-speed optical projection system for dynamic UV-light patterning, and a three-axis computer-controlled stage for X-Y location and focus control. The light from a mercury lamp is filtered to obtain the UV light at a wavelength of 365 nm, which is introduced into the collimating lens device to provide uniform illumination. The DMD chip (Wintech DLP 4100 0.7” XGA, Wintech Digital Systems Technology Corp., Carlsbad, CA, USA) from Texas Instruments plays the role of a mask that reflect the uniform incident UV light pixel-by-pixel to generate image frames. This image will be transferred by the projection objective to a photoresist-coated substrate. The DMD consists of a 1024 × 768 micromirror array with a cell size of 13.68 μm, the demagnification of the projection objective is 6.84. Thus, the theoretical resolution of the UV-light pattern projected on the photoresist surface is 2 μm. The XYZ stage has a travel range of 100 mm × 100 mm in the X-Y plane and 10 mm in the vertical direction with a resolution of 50 nm, which enables us to achieve a large exposure area at the substrate and a precise control of focus.

Figure 1b illustrates the grayscale control based on the pulse width modulation of 8-bit planes, which enable 256 grayscale levels. The single-frame time *T*_0_ of a grayscale image is divided into eight different time intervals controlled by an 8-bit binary sequence. Each micro-mirror unit of the DMD can be individually controlled by a computer in the direction of ±12° (“1” or “0”) to determine whether its working state is ON or OFF in each bit plane. By rapidly (typically 20 μs) changing the rotation direction of the micromirror based on the pulse width modulation technique, we obtain a finely-tuned grayscale level which is in proportion to the time duty cycle of the ON states.

Figure 1c shows the fabrication procedure based on grayscale mask exposure. This procedure consists of two steps: exposure and development. In the first step, the high grayscale level will result in a greater exposure dose distribution on the photoresist surface due to its larger duty cycle time of the ON states. Then, in the second step, the photoresist pattern whose exposure depth is in proportion to the exposure dose will be obtained after development. By using the one-step maskless grayscale lithography, one can flexibly control the grayscale level to modulate the exposure dose required for the fabrication of micro-optic elements with an arbitrary surface.

### 2.2. Nonlinear Effects

Essentially, the aim of generating the grayscale is to control the exposure depth of the photoresist. If the grayscale is of a linear dependence relationship on the exposure depth, then only one scale factor is needed to be calibrated. However, due to the two reasons discussed below, an adjustment curve instead of one scale factor must be determined.

Usually, the exposure dose is defined as the product of the intensity of the incidence light *I*(*x*, *y*) and exposure time *T*:

(1) E(x,y)=I(x,y)×T

**Figure 2:**
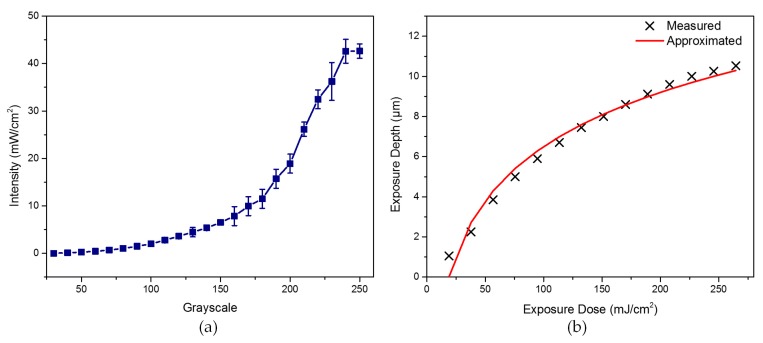
(**a**) Relation between intensity of ultraviolet (UV) light at a wavelength of 365 nm and grayscale levels; and (**b**) the relation between the exposure depth and exposure dose.

In the maskless lithography system, the exposure dose *E*(*x*, *y*) is proportional to the intensity *I*(*x*, *y*) under a pre-setting exposure time. In general, the dependence of the intensity on the grayscale level deviates from the linear relationship due to the peculiarity of the DMD that cannot be systemically eliminated. To estimate the relationship between intensity and grayscale, the intensity data tested by a UV radiation illuminometer (UIT-250, Ushio America, Inc., Cypress, CA, USA) is presented in Table 1, which just gives the data for grayscales over 30 because the intensity below 30 is basically zero. Figure 2a shows an intuitive presentation about this nonlinear relationship. We note that the intensity of a lower grayscale increases slowly at a stable state. In contrast, the intensity of a higher grayscale increases rapidly at an unstable energy level, which is disadvantageous for the control of the surface roughness. There are few specific expressions to describe this nonlinear relationship, but it does exist in the DMD [27]. We assume that this phenomenon may be caused by the nonlinear control of pulse width modulation in Figure 1b. The high level bit planes have larger duty cycle times and contribute a significant intensity, but the low bit plane just has a small duty cycle time and imparts a small intensity. Thus, the final presentation is that the intensity varies exponentially with the grayscale and is unstable at high grayscale levels.

In addition, to test the dependence of the exposure depth on the exposure dose, we performed an exposure test on the photoresist (AZ-9260, Clariant Corporation, Muttenz, Switzerland), which is spin-coated on the substrate at 2000 rpm followed by prebaking at 100 °C for 10 min to obtain a photoresist layer of about 10.5 µm. A grayscale grating of 200 level (18.91 mW/cm^2^) with a 400 μm period that consists of 200 pixels in the horizontal direction is applied to perform this experiment for a convenient measurement. The exposure time changes from 1 s to 14 s which enables a constant exposure dose increment. After development in the developer (AZ 400K, Clariant Corporation, 1:2, 40 s) we extracted the exposure depth by the stylus profiler, as presented in Table 2. Due to the properties of the photoresist, the relationship between the exposure depth and the exposure dose is nonlinear, also. Some studies [28,29] have reported that the exposure depth is a logarithmic function of the dose and can be determined by the contrast curve, which is defined as the linear slope of the contrast curve as follows:

(2) $$\gamma =\frac{1}{\ln E_{\mathrm{cl}}-\ln E_{\mathrm{th}}}=\frac{h\left(x,y\right)/H}{\ln E\left(x,y\right)-\ln E_{\mathrm{th}}},\;\mathrm{where}\;0\text{<}h\left(x,y\right)<H\;\mathrm{and}\;E_{\mathrm{th}}<E\left(x,y\right)<E_{\mathrm{cl}}$$

In Equation (2), the parameter *E*_th_ is the threshold dose to initiate a photoresist reaction, *E*_cl_ is the clearing dose required for removing the photoresist layer *H* completely, and *E*(*x*, *y*) is the required dose for target exposure depth *h*(*x*, *y*), which can be obtained by direct inversion of Equation (2) as follows:

(3) $$h\left(x,y\right)=H\times \gamma \times \ln\left(\frac{E\left(x,y\right)}{E_{\mathrm{th}}}\right)$$

We have calculated the theoretical curve of the exposure depth with respect to the dose. Figure 2b shows the sampling data and theoretical curve about the exposure depth. We note that these two curves are basically approximated. From the above, we know that both the relationship between intensity and grayscale level of the DMD and the relationship between the exposure dose and the exposure depth are all nonlinear.

### 2.3. Grayscale Mask Design

**Figure 3:**
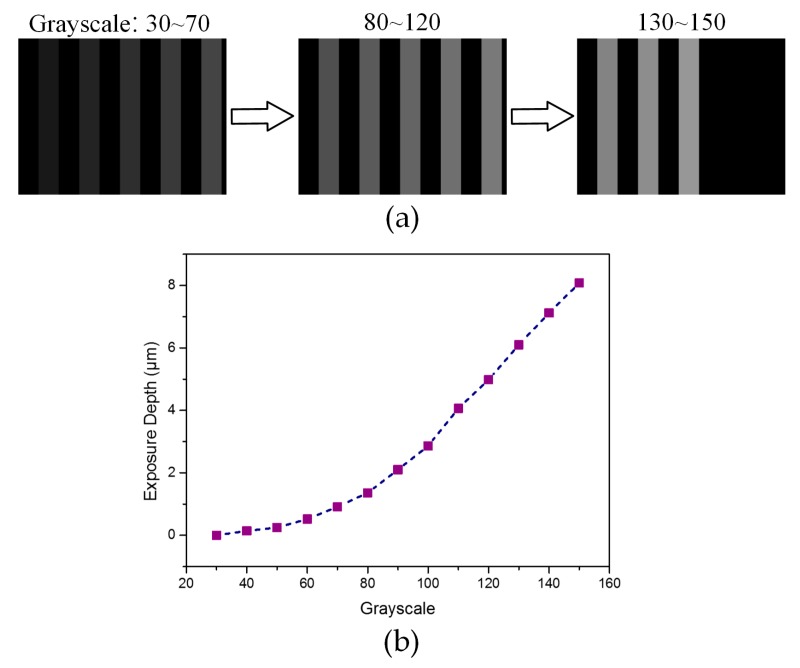
(**a**) Grayscale maps (30 to 150 grayscale levels) of a grating with a period of 400 μm (200 pixels in the horizontal direction) for the calibration; and (**b**) calibration curve between the exposure depth and grayscale level.

To compensate for the two types of nonlinear effects shown in Figure 2, and then generate an accurate grayscale map, a valid method is to find a reliable calibration curve which can provide a precise relationship between the exposure depth and the grayscale value. According to this calibration curve, the grayscale compensation for arbitrary surface designs is achievable. There are two points that deserve consideration in the calibration processing: the calibration curve should maintain smooth-slow growth and a stable intensity level because the rapid increment of the exposure depth and an unstable intensity level will lead to inaccurate control of the surface profile and a terrible surface roughness. Thus, an exposure test was carried out for the grayscale range of 30 to 150 which is a reasonable increment and a stable intensity level according to the curve shown in Figure 2a. The photoresist layer of about 8 μm was prepared at 2500 rpm, which could be flexibly adjusted according to the required thickness. Here we adopt the grayscale maps of a grating to perform this adjustment, as shown in Figure 3a. The period of the grating is set as 400 μm, which consists of 200 pixels in the lateral direction. The exposure time was set as 20 s, which was estimated by the curve shown in Figure 2b to ensure a sufficient exposure dose. The test data measured by the stylus profiler was given in Table 3 and plotted in Figure 3b. This adjustment curve between the exposure depth and grayscale level provides a reference which adequately considers the nonlinear effect between the exposure dose and grayscale level, and the nonlinear effect between the exposure depth and exposure dose, simultaneously.

Since only discrete information about the relationship between the grayscale and the exposure depth can be obtained, in a practical application, to generate a precise grayscale map for a structure to be fabricated, a numerical fitting processing will be adopted. We assume that the exposure depth between two adjacent sampling points is a linear correlation due to the narrow sampling step, thus a linear interpolation method is applied to extracted suitable grayscale level. Although this processing may introduce some errors, it imposes little influence on the generation of the grayscale map due to the average resolution being less than 0.1 μm, which is calculated according to the linear interpolation in a single sample interval.

**Figure 4:**
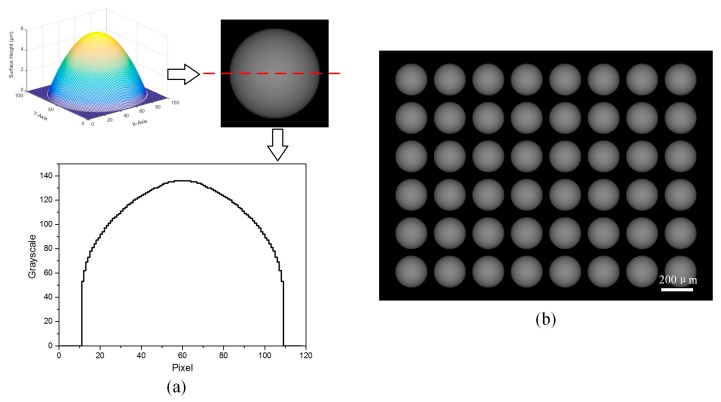
(**a**) Designed spherical micro-lens array (MLA) model and cross-section of the grayscale map of a single lens; and (**b**) grayscale mask with a 6 × 8 lens array.

As an example, we design a micro-lens array (MLA) with a spherical surface (Figure 4a), whose aperture B is 200 μm and height h is 6 μm. This spherical surface can be expressed as:

(4) $$z\left(x,y\right)=\sqrt{r^{2}-x^{2}-y^{2}}-r+h,\mathrm{where}\;0\leq z\leq h\;\mathrm{and}\;0\leq \sqrt{x^{2}+y^{2}}\leq B$$

In the above equation, the parameter r is the radius of this spherical surface and can be numerically calculated according to the aperture and the height. According to Figure 3b, we generate the grayscale map of a single lens which consists of 100 × 100 pixels. From the cross-section of the grayscale map presented in Figure 4a, we note that the variation of grayscale value is stair-stepping. The small steps may impart some influence on the surface roughness, but does not break the outline due to the continuous surface. Finally, the grayscale mask corresponding to this spherical MLA is generated to perform the exposure, as shown in Figure 4b.

## 3. Results and Discussion

**Figure 5:**
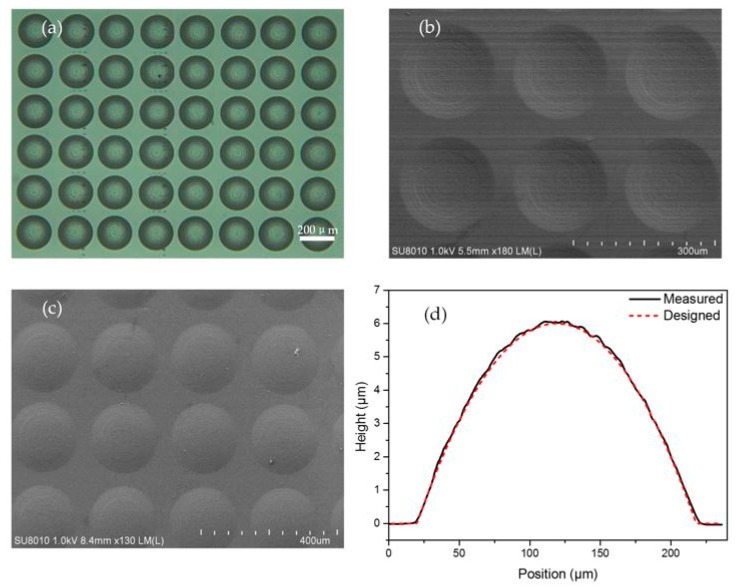
Microscope image (**a**) and scanning electron microscope (SEM) image (**b**) of a concave spherical MLA in photoresist; (**c**) SEM image of a convex spherical MLA in polydimethysiloxane (PDMS); and (**d**) the measured and designed cross-sections of the convex spherical MLA.

To verify the availability of our method, experiment was carried out for grayscale map shown in Figure 4b. For the photolithography, we used positive photoresist (AZ-9260, Clariant Corporation). The photoresist was spin coated on a glass substrate at 2500 rpm followed by a prebaking at 100 °C for 10 min. Then a photoresist film about 8 μm thickness was obtained. Next, the grayscale map was exposed on the surface of photoresist. The total time for the exposure of the grayscale map is 20 s. After development in the alkaline developer (AZ 400K, Clariant Corporation, 1:2) for approximately 40 s, the lens-shaped profile of the photoresist, which corresponding to the reversed profile of the designed model in Figure 4b was obtained. Figure 5a,b shows the microscope and scanning electron microscope (SEM) images of this concave spherical MLA in photoresist.

To obtain the available spherical MLA, the photoresist mold in Figure 5b was reversely transferred in the PDMS elastomer. Here the PMDS Sylgard 184 (from Dow Corning, Midland, MI, USA) was prepared by mixing the PDMS with diluter at a proportion of 10:1 in weight, after which it was kept under vacuum for dehydration for 20 min. Then the PDMS solution was applied to the mold and kept at 100 °C for 15 min. Figure 5c shows the SEM image of a convex spherical MLA on the top of PDMS. To evaluate the surface profile of the PDMS MLA, a cross-section of the convex lens was profiled by the stylus profiler, as shown in Figure 5d. It is obvious that the measurement profile (black solid line) agrees well with the designed profile (red dash line), and the largest difference between them was 0.21 μm (3.5% of the total height). To analyze the deviation between the practical curve and the theoretical curve, we calculated the root-mean-square (RMS) value as follows:

(5) $$R={\left\{\frac{1}{N}\sum_{i=1}^{N}{\left[h\left(i\right)-h_{\mathrm{design}}\left(i\right)\right]}^{2}\right\}}^{1/2}$$

The calculated RMS deviation was 0.08 μm, which was enough for the MLA application in visible light.

**Figure 6:**
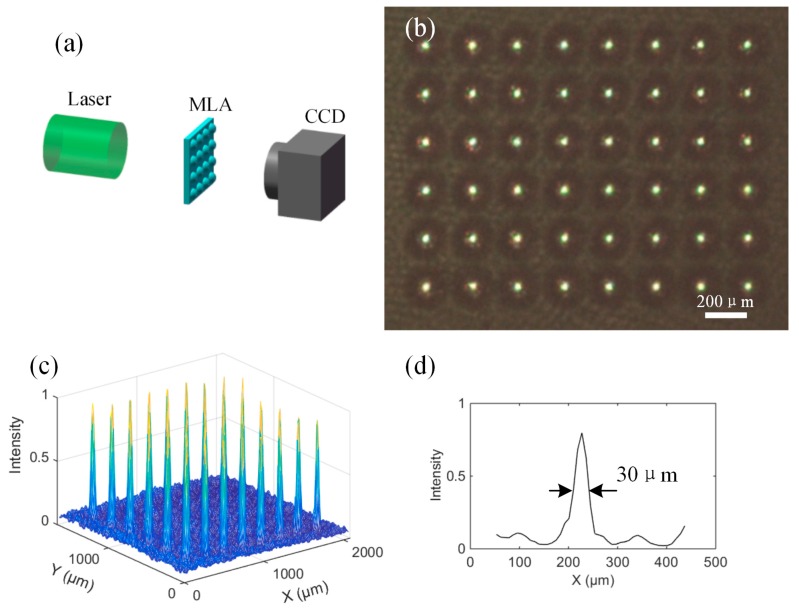
(**a**) Test of the focusing performance of convex spherical MLA in PDMS; (**b**) the image of focused light spots; (**c**) the normalized intensity distribution of focused light spots; and (**d**) an image of the normalized intensity of a single typical single spot.

**Figure 7:**
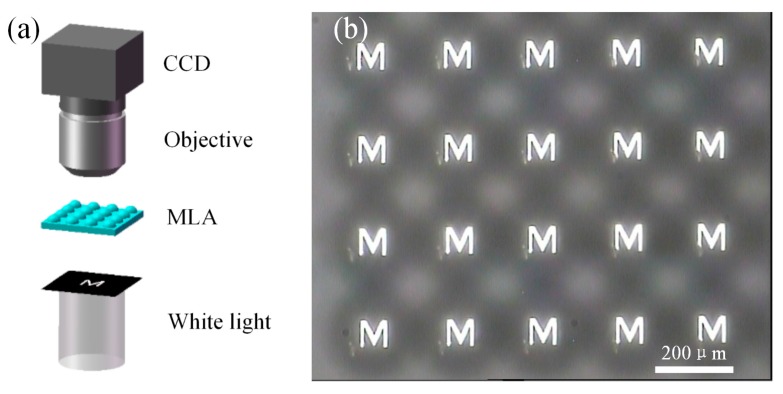
(**a**) Test of the imaging performance of convex spherical MLA in PDMS; and (**b**) the arrayed images of the letter “M” observed by charge-coupled device (CCD).

To estimate the optical performance of the convex MLA in PDMS, both focusing and imaging experiments were carried out, as shown in Figure 6 and Figure 7. In the focusing experiment, a laser beam at a wavelength of 532 nm was introduced to illuminate the whole PDMS MLA. A 6 × 8 light spot array with uniform intensity was captured by a charge-coupled device (CCD) placed in the MLA’s focal plane. Figure 6b,c presents the images of focused light spots and intensity distribution, respectively. Figure 6d shows an image of the normalized intensity of a single typical spot. The full width at half maximum (FWHM) for this particular spot is about 30 μm. The focal length of the MLA was estimated to be about 1.7 mm. Then we tested the imaging ability of the MLA on a microscopy setup. As exhibited in Figure 7, a mask with the letter “M” was placed between the white light source and convex MLA, and the image array was observed by using a CCD camera mounted with an objective lens. The uniform light spots and clear “M” letters indicate that the fabricated convex spherical MLA in PDMS has excellent optical properties, which has important applications in array illumination and micro-imaging.

To further demonstrate that this one-step maskless grayscale lithography is capable with the fabrication of micro-optics with arbitrary surface, we also designed MLAs with an aspherical surface and a conical surface. The expression of the aspheric surface is:

(6) $$z\left(x,y\right)=h\times \exp\left(-\alpha \left(x^{2}+y^{2}\right)/2\right),\mathrm{where}\;0\leq z\leq h,\;\mathrm{and}\;0\leq \sqrt{x^{2}+y^{2}}\leq B$$

and the expression of the conical surface is:

(7) $$z\left(x,y\right)=h\times \left(1-\frac{\sqrt{x^{2}+y^{2}}}{B}\right),\;\mathrm{where}\;0\leq z\leq h,\;\mathrm{and}\;0\leq \sqrt{x^{2}+y^{2}}\leq B$$

**Figure 8:**
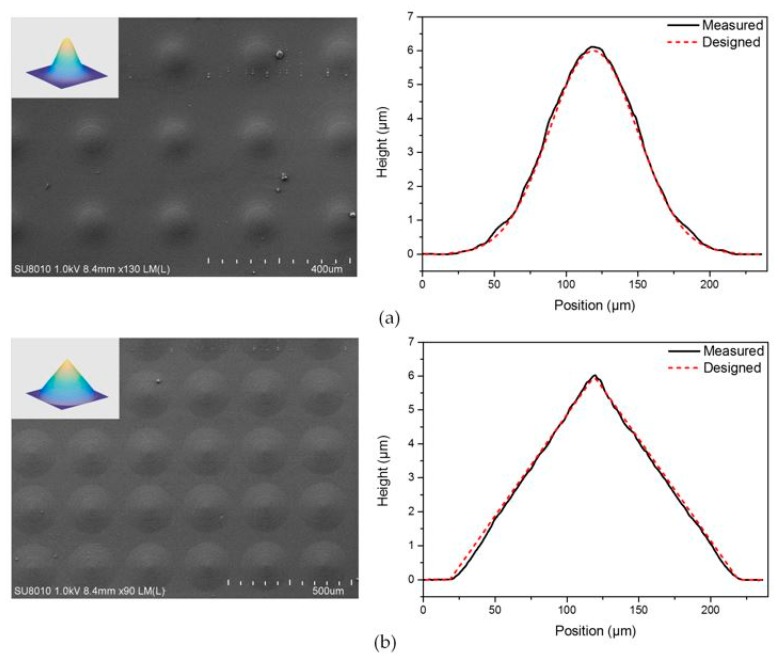
(**a**) SEM image of the aspherical MLA in PDMS and the cross-sections of measurement and design; and (**b**) SEM image of the conic MLA in PDMS and the cross-sections of measurement and design.

In the above equations, the parameters *h*, *α*, and *B* are set as 6 μm, 0.0009, and 200 μm, respectively. Figure 8 shows the fabricated MLAs with an aspherical surface and a conical surface in PDMS. The cross-section of these two kinds of microlenses presented a good agreement with the designs. The RMS deviation of the aspherical and conical microlens were calculated as 0.1 μm and 0.11 μm, respectively, which further indicated that this method enables a precise control of the customized surface profile and is prospective in the fabrication of micro-optics.

## 4. Conclusions

This work reports a one-step maskless grayscale lithography method based on DMD for the rapid and cost-effective fabrication of micro-optical elements with arbitrary surface. The reference curve between the exposure depth and grayscale level effectively compensates the nonlinear effect between intensity and the grayscale level of the DMD, as well as the nonlinear relationship between the exposure depth and dose, and can be used to generate appropriate grayscale map and ensure a precise control of surface profile of the structure to be fabricated. Using this method, we successfully fabricated several typical MLA with a spherical surface, aspherical surface, and conic surface with an aperture of 200 μm and a height of 6 μm in the photoresist. Then these photoresist models were reversely transferred in PMDS for optical testing. The cross-section measurement results agree well with the designed profile. The root mean square (RMS) between the test and design value of the surface height is about 0.1 μm. Additionally, we tested the focusing and imaging ability of the replicated convex spherical MLA in PDMS. Both the uniform focus light spots and clear image of the letter “M” indicated that the generated MLA in PDMS could achieve excellent optical properties. These results demonstrate that the proposed method of compensating the nonlinear effect in maskless lithography can be directly used to control the grayscale levels of the DMD for fabricating the structure with an arbitrary surface profile.

## Figures and Tables

**Table 1:** The tested data between intensity and grayscale level.

Grayscale	Intensity (mW/cm^2^)	Standard Deviation (mW/cm^2^)	Grayscale	Intensity (mW/cm^2^)	Standard Deviation (mW/cm^2^)
30	0	0	150	6.51	0
40	0.13	0	160	7.85	0.02
50	0.26	0	170	9.95	0.02
60	0.43	0	180	11.5	0.02
70	0.7	0	190	15.73	0.02
80	1.03	0	200	18.91	0.02
90	1.5	0	210	26.15	0.015
100	2	0	220	32.46	0.02
110	2.78	0.005	230	36.22	0.04
120	3.63	0.005	240	42.6	0.025
130	4.47	0.01	250	42.63	0.015
140	5.37	0.005	-	-	-

**Table 2:** The tested exposure depth data under different exposure doses.

Exposure Dose (mJ/cm^2^)	Exposure Depth (μm)	Standard Deviation (μm)	Exposure Dose (mJ/cm^2^)	Exposure Depth (μm)	Standard Deviation (μm)
18.9	1.05	0.025	151.2	8	0.25
37.8	2.25	0.2	170.1	8.6	0.23
56.7	3.85	0.1	189	9.12	0.19
75.6	5	0.15	207.9	9.6	0.18
94.5	5.9	0.15	226.8	10	0.15
113.4	6.7	0.2	245.7	10.26	0.18
132.3	7.45	0.25	264.6	10.53	0.19

**Table 3:** The exposure depth measurement result under a 20 s exposure time.

Grayscale	Exposure Depth (μm)	Grayscale	Exposure Depth (μm)
30	0	100	2.86
40	0.14	110	4.06
50	0.25	120	4.98
60	0.52	130	6.1
70	0.91	140	7.12
80	1.36	150	8.08
90	2.1	-	-
